# Contextualising sex and gender research to improve women's health: An early- and mid-career researcher perspective

**DOI:** 10.3389/fgwh.2022.942876

**Published:** 2022-07-19

**Authors:** Laura Hallam, Briar L. McKenzie, Jessica Gong, Cheryl Carcel, Carinna Hockham

**Affiliations:** ^1^The George Institute for Global Health, University of New South Wales, Sydney, NSW, Australia; ^2^Australian Human Rights Institute, University of New South Wales, Sydney, NSW, Australia; ^3^The George Institute for Global Health, Imperial College London, London, United Kingdom

**Keywords:** sex, gender, women's health, equity, research methods

## Abstract

The field of sex and gender research in health and medicine is growing, and many early- and mid-career researchers (EMCRs) are developing skills in this area. As EMCRs specialising in sex and gender research, we aim to better understand sex- and gender-based determinants of human health, challenge long-standing and pervasive gender biases, and contribute to improving the evidence base upon which clinical guidelines and policy interventions are developed. To effectively achieve these goals, we believe that EMCRs would benefit from understanding the challenges of working in this space and participate in driving change in three key areas. First, in creating greater links between the goals of sex and gender research and addressing systemic bias against women and gender minorities, to effectively translate knowledge about sex and gender differences into improved health outcomes. Second, in expanding the reach of sex and gender research to address women's health in an intersectional way and ensure that it also benefits the health of men, transgender and gender-diverse people and those who are intersex. Third, in working with others in the scientific community to improve methods for sex and gender research, including updating data collection practises, ensuring appropriate statistical analyses and shifting scientific culture to recognise the importance of null findings. By improving focus on these three areas, we see greater potential to translate this research to improve women's health and reduce health inequities for all.

## Introduction

There is a well-acknowledged bias against, and underrepresentation of, women in health and biomedical research (1, 2). To address this, there have been many calls for greater consideration of sex and gender in research, a movement that has gained traction over the last 10 years (3–6). This research has contributed to an emerging evidence base that demonstrates the impact of sex and gender on the presentation, diagnosis, treatment and outcomes of many diseases, including major chronic diseases, as well as health behaviours and access (7). This field of research has strong links to women's health, and feeds into new approaches to women's health that address health across the life course, creating links between reproductive health, pregnancy management and chronic disease in women (8). What has followed is a new generation of researchers focused on sex- and gender-sensitive research and who are interested in what this work means within the broader context of health inequities. Here, we discuss sex and gender research as an emerging trend in global women's health and summarise what we see as important considerations for the future of the field, including the applicability of sex and gender research beyond women's health. We reflect on the lessons we have learnt as early- and mid-career researchers (EMCRs) as we navigate the complexities of this area of work and on the challenges we face, including bringing new ways of thinking into established research systems and navigating diverse perspectives on sex, gender and health. In this Perspective, we use the definitions of sex and gender as described by Tannenbaum et al. (4).

In conducting our own research, encompassing different therapeutic areas and sex and gender policies, we visualise sex and gender research as situated inside a complex system including: (i) wider systemic issues of gender bias and health inequities, (9) (ii) greater recognition of gender diversity and intersectional perspectives, (10–12) and (iii) efforts to improve research methodology and rigour (4, 13, 14).

### Conducting and disseminating sex- and gender-based research in the context of systemic gender bias

Sex and gender research generally aims to expose, understand, and address health inequities. In particular, this research area focuses on the underrepresentation of women in research studies and the gaps in our understanding of the impact of sex and gender on health and disease. However, researchers, including ourselves, are investigating sex and gender, and their relationship with disease, in systems that reflect broader society's biases against women and gender minorities. This has consequences for the effective translation of research findings into sex- and gender-sensitive approaches to reduce health disparities, and so it is equally important to consider and address gender bias in other areas.

First, we must consider that our research is being disseminated to readers that may hold biassed views on women and gender minorities. As a result, there is the potential for findings from sex and gender research in health to be misinterpreted, by researchers, the public and the media (15, 16). In particular, there is a risk of losing nuance and defaulting to biological essentialist explanations for observed differences, (15, 16) which can have the unintended consequence of reinforcing patriarchal hierarchies, rather than challenging them to support health equity. It is therefore critical to ensure that, when planning, disseminating and translating research about sex and gender differences in disease, the research is firmly linked to the goal of addressing bias and reducing health inequities, and clearly articulated as such.

Second, we should consider how sex and gender research can be used to support wider systemic changes, such as increased women's rights, better access to healthcare and education, and freedom from violence and structural discrimination (9). This can include research translation to achieve the United Nations Sustainable Development Goals (SDGs), including those for better health and gender equality (17). Without addressing these wider systemic changes and considering the variations in systemic barriers and discrimination based on geography and culture, it will be difficult to improve women's health. Achieving this goal will require the breaking down of siloes between research disciplines. Effectively understanding the impacts of both sex- and gender-related characteristics on health, and how these link into societal power structures, requires multidisciplinary knowledge across biomedicine, public health and social sciences that would be difficult for any single researcher to acquire. Innovative multidisciplinary research can support the creation of these links and assist in developing tools to better account for multisectoral factors (18). For example, population ageing is one of the greatest challenges in the 21^st^ century, with 1 in 6 people expected to be 60 years and older by 2030 (19). The ageing experience can be wide-ranging, complex and unequal, and has various health-related impacts while also reflecting structural inequities, many of which are gendered (18, 20). A recent publication assessed five domains of societal ageing (wellbeing, productivity and engagement, equity, cohesion, and security) and conducted a gendered analysis, which showed advantage for men across all domains in all the countries studied, despite women having longer lifespans (21). This innovative research combined analysis of health and functional ability with societal and structural dimensions that impact ageing, providing a more nuanced understanding of sex and gender differences in ageing, and highlighting a range of intervention points to address them.

Third, incorporating sex- and gender-based findings into clinical guidelines, and implementing these guidelines into clinical practise, is subject to gender bias amongst health professionals and review processes. Gender bias in clinical and social care is a major contributor to poorer health outcomes in women, (2) and while these disparities can be revealed by sex- and gender-disaggregated research, solutions are needed to address the bias. Partly, sex and gender research can achieve this by directly challenging equivalencies between women's health and reproductive health, and drawing attention to women's experiences of non-sex-specific diseases that are often considered, without nuance, to be more prevalent or serious in men, such as heart disease (22). Other ways to address bias include improving medical education, dispelling hysteria myths, having sex-specific clinical guidelines (where appropriate), and trusting women to be reliable narrators of their own health experiences (2). Systems and tools that directly address bias in clinical interactions should also be explored. For example, a recent paper outlined the potential for artificial intelligence to be used to improve cardiovascular disease (CVD) screening in women, (23) which could operationalise the extensive knowledge base of sex and gender differences in CVD as well as address bias in healthcare professionals' decisions around diagnosis and treatment (24).

Fourth, the lack of women in leadership across science and medicine has an impact on the effective translation of this work into women's health agendas, funding opportunities and publication in high-impact journals. Global Health 50/50's 2021 report showed that, while most front-line health workers and junior researchers are women, 70% of leadership positions in global health organisations are held by men (25). A 2021 study found that women represent only about 1 in 5 editors in chief of top-ranked medical journals (26). As it has been clearly demonstrated that women authors are more likely to account for sex and gender in their work, (27) challenging gender inequalities in the scientific and health workforce can help address issues holistically across the sector. Initiatives such as Global Health 50/50 (25), the World Health Organisation's gender mainstreaming approach (28) and the Association of Australian Medical Research Institutes' Gender Equity, Diversity and Inclusion Strategy and Action Plan (29) provide frameworks for this multi-pronged approach.

Lastly, research into sex and gender differences can bolster the development of FemTech, defined as technologies that assist women's health. However, women leading the translation of this knowledge into innovative technologies face challenges with investment. Only 2% of venture capital funding is invested in women-led companies and only 0.64% for women of colour (30). Despite these barriers, the FemTech sector is expected to grow significantly in coming years (30).

### Broadening the focus of sex and gender sensitive research, without losing the link to women's health

Focus on sex and gender in research has had strong links to women's health, with advocates asking for better representation of women in research, (31) removal of discriminatory exclusion laws, (32) improved focus on female-specific health issues and a movement away from the “default male” practise of medical research (33). From this, the field has come to focus on identifying disparities in women's and men's (binary) experiences of disease. Indeed, while there has been a particular focus on identifying inequities that negatively impact women, sex and gender research also allows for areas where men are disadvantaged to be identified. In recent years there has been increasing discussion of the need to expand this field beyond a comparison of women and men and to improve definitions and understanding of sex and gender to include the experiences of transgender, gender-diverse and intersex people (12, 34). Advocating for improved understanding of sex and gender concepts, including the difference between the terms, consolidates understanding of the diverse biological, sociocultural and environmental influences that shape women's health as well as promoting more inclusive science that recognises diverse identities.

Sex and gender research is approached in many ways, with some focusing on sex differences, some on gender differences and other work investigating the impact of sex- and gender-related factors on health (35). In many areas of the field, there has been a prioritisation of the role of biological sex over gender, influenced by policies such as the US National Institutes of Health's Sex as a Biological Variable (SABV) policy (36). While sex can be more straightforward to break down into specific sex characteristics and variables of interest, understanding the impact of gender, including identity, roles, norms and relations, can be more complex, but is essential (37). Londa Schiebinger's group from Gendered Innovations (38) developed a Gender as a Sociocultural Variable tool as a complement to SABV, to provide gender-related variables for health researchers to use in their studies (39). The Canadian Centre for Gender and Sexual Health Equity has created a toolkit to encourage researchers to interrogate the aspects of sex and gender they are interested in examining, in order to broaden inclusion criteria for research studies to encompass transgender, gender-diverse and intersex people (40). These tools and frameworks also have value in encouraging researchers to map the complexity of sex- and gender-related characteristics, challenge default biological assumptions and acknowledge diversity of experiences influencing health and disease (35).

People do not experience inequities separately, and sex and gender research as a whole must be conducted alongside considerations of the influence and interactions of other biological and sociodemographic factors, such as age, race and ethnicity, geography, migration status, and socioeconomic status. Intersectionality frameworks can be integrated to facilitate research that is designed to improve health outcomes more equitably (10, 11). This builds on the work of advocates aiming to fill gaps in knowledge about women's health by ensuring that, while men may not be the default human in future research, neither should straight, white, cis-gendered women be deemed to represent all women nor Western gender norms and power structures considered to be generalisable to other contexts. Together with better community consultation, diversity amongst both researchers and research participants is going to be needed if we want a research ecosystem that better serves the needs of the population and does not perpetuate stereotypes or increase stigma.

### Effective collection, analysis and reporting of data

A sex- and gender-sensitive approach to the collection, analysis and reporting of health data is central to driving improvements in sex- and gender-based health inequities. However, most human studies continue to treat sex and gender as synonymous, whilst confusion around the use of these terms has also been reported for studies in non-human animals (14). For research involving primary data collection, researchers should consider whether data on a participant's sex, gender, or both, is needed to answer the question in hand. More than two options should be provided for both sex and gender questions and participants should be given the opportunity to self-identify (41). For studies using existing datasets, it is important to be cognizant of the limitations of the data, including whether sex or gender data are reported, whether transgender, gender-diverse and intersex people are captured by the data collection methods used, and the availability of sex-specific or gendered variables for analyses. The ambiguity with which sex and gender are reported in existing datasets and published literature is a challenge that we frequently face in our own research. Development of standardised sex and gender data collection and reporting tools, validated in different parts of the world and in consultation with community and health advocacy groups, will help reduce uncertainty around which variables are collected, and how. Crucially, developing inclusive methods will assist in avoiding perpetuating the idea that some people are too difficult to include in research, an accusation that was once, and still is, levelled at women (42).

Where feasible, studies should be designed with adequate power to detect sex or gender differences, whether it be with sex/gender as the main exposure of interest or as a potential effect modifier, and this should be specified *a priori*. For the latter, this will typically require a larger sample size than if sex or gender were simply treated as a confounder (as is typically done). The need for large sample sizes in sex and gender research is why large-scale population databases such as the UK Biobank, (43) and routinely-collected datasets such as the Medicare Benefits Schedule in Australia, (44) are so invaluable, despite not collecting sex and gender separately, and why a move towards more gender-inclusive data collection in datasets like these should be treated as a priority. Programmes like Our Future Health in the UK, (45) which is being set up to create a diverse and inclusive cohort that better represents the UK population, will be extremely valuable to the field.

Analyses of sex or gender differences in exposure-outcome associations requires more than simply splitting the data into the sexes/genders and reporting findings for each group separately. However, a recent review on animal and human studies found that 45% of articles claiming to have identified sex-specific treatment effects had done precisely that and incorrectly stated that a sex difference was present due to different conclusions being drawn for each sex (14). As with any estimation, differences in effects between subgroups are subject to sampling error and so formal testing for whether these differences could have arisen by chance is important (46). The review found that only 29% of studies explicitly tested for an interaction between sex and the exposure of interest. Whilst in-depth discussion of correct statistical approaches to evaluating sex and gender differences is beyond the scope of this perspective, we refer the readers to an excellent tutorial paper on this topic (46). Training in appropriate statistical methods to identify sex and/or gender differences is essential for EMCRs conducting quantitative work.

Where large sample sizes are not appropriate or feasible, or researchers are not undertaking their own sex and gender analysis, results should still be provided disaggregated by sex or gender so pooling of data into a meta-analysis can be performed. Currently this is infrequently done, as demonstrated by COVID-19 reporting, with less than half of countries reporting key COVID-19 metrics by sex or gender (47). Additionally, the absence of a difference should not be considered a null finding, with findings of similarities between women and men having the potential to counteract biassed or stereotyped beliefs about sex and gender. For example, when it comes to assessing and recording dietary intake data there has been suggestions based on findings from qualitative reviews and some studies that suggest women are more likely to underestimate what they eat in comparison to men, likely due to societal pressure and the influence of diet culture that tends to target women. However, in a meta-analysis conducted by this group, we found that the degree of underestimation was similar for women and men, across a range of diet assessment methods used (48). This null finding has been important in “de-bunking” a perception that women are poor reporters of dietary intake. It is also an important finding for research into sex and gender differences in the diet related burden of disease, as it makes it less likely that differences in the diet disease burden are just due to reporting differences.

## Discussion

Sex and gender research is evolving, and while it is exciting to be EMCRs in this space, it is also complex and challenging. We, and likely many others, have ended up working in this field due to our interest in both science and gender equity and are joining decades of advocacy to ensure that science is conducted in a more equitable way that effectively captures the experiences and meets the needs of women. As the next generation of researchers, we have the potential to be influential in changing practise to support and expand on these goals. None of us in our individual work can overcome all of the challenges and limitations or address all the systemic issues we discuss in this perspective, but we can strengthen the field by creating links between our own work and broader, multidisciplinary research and wider advocacy efforts. We have the opportunity to work together, as well as with clinicians and innovators, to collectively advance our knowledge, improve our research practise and advocate for others to do the same. Further, we have the guidance of many in this field who have put together invaluable resources to support new researchers who are learning about sex, gender, science, and health, including Gendered Innovations (38) and the Canadian Institutes of Health Research (49). Through our own collaborations, we have been able to add to the collections of resources available to support health and medical researchers to understand how to integrate sex and gender considerations into their work (50, 51).

From reflecting on our own experiences and recent research, we see scope for three defined areas to be focused on, and strengthened, in years to come: translating sex and gender research in biassed systems, aiming for greater inclusivity, and improved research methods (Table 1).

**Table 1:** Focus areas for the future of sex and gender research.

**Focus area**	**Key points**
Conducting and disseminating sex- and gender-based research in the context of systemic gender bias	Link sex and gender research to the goal of improving health equity
	Understand links between sex and gender research and efforts to create wider systemic change to improve women's health
	Address gender bias in clinical care to support effective translation of sex and gender research
	Support women to achieve leadership positions in science, health and medicine to create cultural change in the sector
	Support women innovators to address women's health issues
Broadening the focus of sex and gender sensitive research, without losing the link to women's health	Improve understanding of sex and gender concepts to improve health for all
	Increase focus on the impact of gendered factors, as well as sex
	Use intersectional frameworks to understand impact of varied sociodemographic factors on health
Effective collection, analysis and reporting of data	Aim to collect inclusive sex and gender data or acknowledge the limitations of previous data collection
	Aim to design studies with adequate power to detect sex and/or gender differences
	Use appropriate statistical methods to determine presence of sex and/or gender differences
	Report null findings

We believe that improving women's health requires greater contextualising of sex and gender research within this system, and that this is an exciting and challenging evolution for EMCRs to contribute to. Such a focus will lead to advancements towards gender equity, more inclusive science, and better-quality research, to contribute to equitable improvements in health outcomes globally.

## Data availability statement

The original contributions presented in the study are included in the article/supplementary material, further inquiries can be directed to the corresponding author.

## Author contributions

LH, BM, JG, and CH had the idea for this perspective. LH wrote the first draft. LH, BM, JG, CC, and CH reviewed, edited, and contributed to writing the following versions of the manuscript. All authors critically reviewed and approved the final copy of the manuscript.

## Funding

LH was funded by an anonymous philanthropic donor. CC was funded by the National Heart Foundation of Australia (Postdoctoral fellowship 102741) and National Health and Medical Research Council of Australia (NHMRC) investigator grant (APP2009726).

## Conflict of interest

The authors declare that the research was conducted in the absence of any commercial or financial relationships that could be construed as a potential conflict of interest.

## Publisher's note

All claims expressed in this article are solely those of the authors and do not necessarily represent those of their affiliated organizations, or those of the publisher, the editors and the reviewers. Any product that may be evaluated in this article, or claim that may be made by its manufacturer, is not guaranteed or endorsed by the publisher.
